# Simple and noninvasive method for assessment of digestive efficiency: Validation of fecal steatocrit in greenfinch coccidiosis model

**DOI:** 10.1002/ece3.2575

**Published:** 2016-11-17

**Authors:** Richard Meitern, Mari‐Ann Lind, Ulvi Karu, Peeter Hõrak

**Affiliations:** ^1^ Department of ZoologyTartu UniversityTartuEstonia; ^2^ Department of Biology IILudwig‐Maximilians‐University MunichPlanegg‐MartinsriedGermany

**Keywords:** acid steatocrit, *Carduelis chloris*, Coccidiosis, digestion, fat absorption, immunoecology, intestinal health, steatorrhea

## Abstract

1. Animals’ capability to absorb energy and nutrients from food poses a major internal constraint that affects the amount of resources available for allocation to maintenance, growth, signaling, and reproduction. Intestinal surface is the largest area of contact between immune system and microbial antigens; gut thus appears the main arena where trade‐offs between immune function and other components of fitness arise. Assessment of the integrity of digestive machinery should therefore be of high priority in ecophysiological research. Traditional methods of digestive physiology, however, appear unsuitable for most ecological applications due to lethality or complexity of the procedure.
2. Here, we test the reliability of a simple, cheap, and noninvasive procedure, an acid steatocrit that assesses fat content in feces. It is based on centrifugation of a fecal sample, diluted in acid medium, in hematocrit capillary tube and quantifying the percentage of fat in fecal matter. The method has been previously validated in humans and mice; here, we apply it for the first time in birds.
3. When applied to captive wild‐caught greenfinches, the method showed reasonable internal consistency (*r*_s_ = 0.71 for steatocrit values, sampled from the same fecal aliquot in duplicate but processed separately). Individual steatocrit values were significantly repeatable in time in different intervals from eight to at least 20 days (*r*_s_ = 0.32–0.49). The relationship between intestinal health and steatocrit values was tested by experimental manipulations. Medication against coccidiosis (a naturally pervasive intestinal infection) reduced, and experimental infection with heterologous coccidian strains increased steatocrit. Individual changes in steatocrit correlated negatively with changes of two markers of nutritional state—plasma triglyceride levels and body mass.
4. Findings of this study suggest that steatocrit has a wide application potential as a marker of intestinal health in ecophysiological research. In particular, we see the perspective of this method for increasingly popular immunoecological research, conservation medicine, and studies of animal coloration.

## 1 Introduction

Acquisition and processing of energy, that is, feeding and digesting, play a major role in the life of all animals. Not all of the food that is eaten can be utilized and is available for metabolism due to the inefficiencies in the digestive process. Animals’ capability to extract energy and nutrients from food thus poses a major internal constraint on energy processing. Such internal constraints can largely explain why individuals differ in their energy management and allocation decisions (reviewed by Bairlein, 1999; Becker, Encarnação, Kalko, & Tschapka, 2012; Elliott et al., 2014; Karasov & Del Rio, 2007).

Digestion is important to ecologists also for other reasons. Digestive organs, given their high metabolic activity and cell turnover rates, contribute importantly to individual variation in metabolic rates (Dibner & Richards, 2005; Killpack & Karasov, 2012) which eventually determine organisms’ maximum aerobic capacity. Precursors of pigments used for signaling and crypsis are absorbed from food, so that expression of animal coloration can provide information on the integrity of digestive machinery (Hill, 2006; Roulin, 2009). Finally, intestinal surface is the major contact zone between immune system and food‐borne and microbial antigens as the vertebrate gut is home to one of the most densely populated microbial populations on earth (Ley, Peterson, & Gordon, 2006). Functioning of the digestive system therefore provides information about the animals’ capability to resist infections. To study all these topics, ecologists need feasible proxies for assessment of digestive efficiency in wild animals.

One of such simple, cheap, and noninvasive techniques comprises gravimetric assessment of fat content in feces. The method was initially developed as a quick technique for diagnosing fat malabsorption in 5‐day‐old infants in India (Phuapradit, Narang, Mendonca, Harris, & Baum, 1981). After implementation by Tran et al. (1994), the method became known as an acid steatocrit. It is based on centrifugation of a fecal sample, diluted in acid medium, in hematocrit capillary tube and quantifying the percentage of fat in fecal matter. Both fresh and frozen samples can be used.

The method has been validated in human studies, showing high correlations (*r* = 0.8) with actual fat concentration of feces (Amann, Josephson, & Toskes, 1997; Sugai et al., 1994; Tran et al., 1994; Van den Neucker et al., 1997) and fecal energy content [*r* = 0.8, Van den Neucker et al. (2002)]. Steatocrit also shows high specificity and sensitivity for diagnosing fat malabsorption (Sugai et al., 1994; Van den Neucker et al., 1997), villous atrophy (Iacono et al., 1991) and pancreatic insufficiency in cystic fibrosis (Tardelli, Camargos, Penna, Sarkis, & Guimarães, 2013). Infants diagnosed for infectious enteritis caused by *Salmonella typhimurium*,* Klebsiella* spp., and rotavirus had elevated steatocrits (Carroccio, Montalto, Cavataio, & Iacono, 1996). In infants recovering from necrotising enterocolitis, steatocrit significantly predicted daily weight gain [*r*_s_ = −0.71, Rawashdeh, Lloyd, Puntis, Brown, and Booth (1992)]. This method has been also successfully applied for studies of digestion in mice (e.g., Takahashi et al., 2007; Weidemann et al., 2015).

The purpose of this study was to examine the reliability of the steatocrit measure and to apply it for the first time in birds, favorite study objects of immunoecological research (e.g., Hasselquist, 2007). First, we aimed to test whether this is a reliable technique as to enable to detect between‐individual differences in fat absorption capacity that are persistent (repeatable) in time. Second, does this technique adequately reflect experimental manipulations expected to change fat absorption? The rationale of our approach is based on an assumption that individuals differ in their ability to digest fat and that this ability is related to various microbial infections that can be manipulated by experimental infection and antimicrobial treatments. This assumption is largely based on previous work on wild‐caught captive greenfinches (*Carduelis chloris*), our model system.

Greenfinches like other passerines with similar biology regularly suffer outbreaks of digestive tract infections such as salmonellosis (Giovannini et al., 2013; Grant, Todd, & Pennycott, 2007; Lawson et al., 2010), trichomonosis (Lehikoinen, Lehikoinen, Valkama, Väisänen, & Isomursu, 2013) and coccidiosis (Hõrak et al., 2004). All these microbes inhibit the absorption of nutrients, so we expected that experiments aiming at manipulation of infection will affect fat absorption capacity, estimated on the basis of steatocrit.

In order to manipulate diverse components of intestinal microbiota, we treated greenfinches by administration of three different antimicrobials and also performed experimental coccidian infection. One of the antimicrobials—toltrazuril—is designed specifically for treatment of coccidiosis and lacks known effects on microbes other than apicomplexans (see Sepp et al., 2012). Metronidazole has wider spectrum of activity against anaerobic bacteria and protozoans and is widely used for medication of trichomonosis (Samuelson, 1999). Sulfadimethoxine is a broad‐spectrum sulfonamide drug of antiprotozoal (including anticoccidial) and antibacterial efficacy (Mitrovic & Bauernfeind, 1971).

All the greenfinches in our study population have been naturally infected by isosporan coccidians (Sepp et al., 2012), and occasionally, some birds in the aviary have died with symptoms characteristic to trichomonosis (Männiste & Hõrak, 2014). Both toltrazuril (Sepp et al., 2012) and sulfonamide drug Vetacox (Hõrak et al., 2004) reduce the intensity of coccidian infection; birds treated with sulfonamide have higher plasma triglyceride levels (a marker of nutritional state) and body mass than birds experimentally infected with coccidians (Hõrak et al., 2004). We thus predicted that (i) treatments with toltrazuril and sulfadimethoxine result in decrease in steatocrit values as compared to untreated control birds. We have also shown that infecting greenfinches with novel coccidian strains originating from multiple hosts cause decline in body mass and plasma triglyceride levels (Hõrak, Saks, Karu, & Ots, 2006). Hence, we predicted that (ii) such experimental infection will result in increase in steatocrit as compared to untreated control birds. We also predicted that (iii) if any of the microbes that are sensitive to metronidazole cause fat malabsorption, we will see decline in steatocrit among metronidazole‐treated birds. Additionally, we asked whether coccidian infection intensities correlate with steatocrit at individual level and whether individual steatocrit values correlate with body mass and plasma triglycerides. Detection of strong correlations between steatocrit vs intensity of coccidiosis, plasma triglyceride content and body mass would confirm that inhibition of fat absorption by intestinal parasites appears a major pathway affecting nutritional state under current experimental conditions.

## 2 Methods

### 2.1 Study protocol

**Figure 1:**
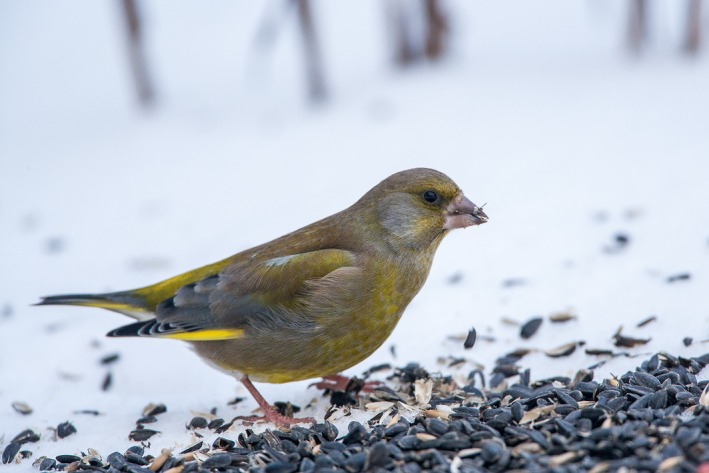
Male greenfinch at winter‐feeding site where intestinal infections likely spread due to contamination of food with feces and saliva. Photograph by Arne Ader, http://www.loodusemees.ee/en/picture-library

Wild greenfinches (Figure 1, 70 males and 41 females) were captured in mist nets at bird feeders in a garden in the city of Tartu, Estonia (58°22′N; 26°43′E) on 5th, 6th, and 8th January 2015. The birds were housed indoors in individual cages (27 × 51 × 55 cm) with sand‐covered floors in a single room where they had visual contact with their neighbors. The average temperature in the aviary during the experiment was 15.5 ± 1.0° (*SD*) °C, and average humidity was 57 ± 7 (*SD*) %. The birds were supplied *ad libitum* with sunflower seeds and tap water and were exposed to a natural day‐length cycle using artificial lighting by luminophore tubes. They were released back to their natural habitat on 3rd (males) and 23rd (females) March 2015. The study was conducted under license from the Estonian Ministry of the Environment (Licence # 1‐4.1/11/100, issued on 23rd March 2011), and the experiment was approved by the Committee of Animal Experiments at the Estonian Ministry of Agriculture (decision # 95, issued on 17th January 2012).

**Figure 2:**
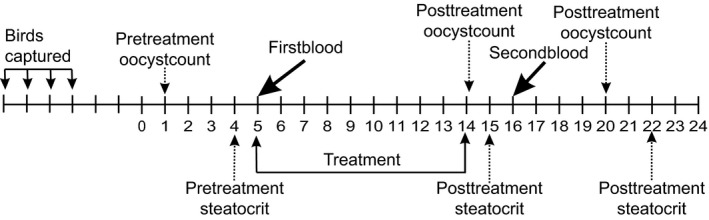
Timeline of the experiment with male greenfinches. Day 1 = 15th January

Males and females received different experimental treatments because we were interested in testing the effects of three different types of antimicrobials and wanted to keep the models simple (i.e., to avoid the possible sex × treatment interactions). Timeline of the experiment on males is shown in the Figure 2. Fecal samples for assessment of pretreatment coccidian infection intensity were collected in the evening of day 1 (15th January) and for measurement of steatocrit on day 4. Birds were weighed and blood sampled in the morning of the day 5. Thereafter, they were divided into three approximately equal‐sized groups on the basis of similar age composition (yearlings vs. older, determined on the basis of plumage characteristics), body mass, and coccidian infection intensity, recorded on the day 1. On the evening of day 5, the birds in two groups subjected to medication treatment started to receive either toltrazuril (24 birds) or metronidazole (23 birds) with their carotenoid‐enriched drinking water. Twenty‐three control birds (one of which died on day 4) received just carotenoid‐enriched water. Birds in the anticoccidial medication group received 2 ml/L solution of Intracox Oral (Interchemie, Castenary, the Netherlands), containing 25 mg/L toltrazuril. Metronidazole (Fresenius Kabi Polska, Kutno, Poland) was administered in concentration of 400 mg/L. Both drugs were dissolved in carotenoid solution (1 ml/L mix of lutein and zeaxanthin (20:1, w/w), prepared from OroGlo brand 15 Liquid Pigmenter with 15 g/kg xanthophyll activity (Kemin AgriFoods Europe, Herentals, Belgium)). Carotenoids were added to drinking water to compensate for naturally low carotenoid content of sunflower seeds. Medication lasted for 10 days, and carotenoid supplementation lasted until the birds were released.

All males were weighed and blood sampled on days 5 and 16 in order to record the effects of treatments on hematological parameters. Blood sampling of birds took place in the mornings before the lights turned on. Other procedures, including fecal sample collection and maintenance, were performed in the evenings after the lights had turned off.

Fecal samples for determination of coccidian (*Isospora* sp.) infection intensity were collected from all the birds on days 1, 14, and 20 and for measurement of steatocrit on days 4, 15, and 22. For collection of fecal samples, two sheets of A4 paper were placed on the sand bedding of cages 2 hr before the lights turned off. After the lights had turned off, feces were collected from the papers. Infection intensities (number of oocysts per gram of feces) of individual birds were quantified as described by Hõrak et al. (2006). All birds appeared naturally infected. Plasma triglyceride concentration was determined from 2.5 μl samples by the GPO‐PAP method (Human GmbH, Wiesbaden, Germany).

Experimental treatment of females involved medication with Sulfadimethoxine (14 birds) and infection with unfamiliar coccidian strains (14 birds); 13 females served as controls. On the 54th day of the study (9 March), fecal samples were collected for determination of pretreatment steatocrits and on day 56, for determination of coccidian infection intensity. All the birds were blood sampled and weighed in the morning of the day 58. Thereafter, they were divided into three groups on the basis of similar age composition, body mass, and coccidian infection intensity. In the evening of day 59, a dose of 2000 sporulated oocytes, diluted in 1 ml tap water, was administered orally by micropipette to birds in the experimental infection group. Excreted oocytes for inoculation were collected from 20 male birds during 3 days. Fecal samples were maintained in 2% potassium dichromate solution at room temperature and aerated daily. Inocula of sporulated oocytes for experimentally infecting were prepared as a single stock from all donor individuals as described by Hõrak et al. (2004). From the evening day 59, birds in the medication group started to receive Sulfadimethoxine (Sigma PHR1448) 250 mg/L, dissolved in carotenoid solution. The treatment lasted for 7 days, that is, until day 65. All females received the same carotenoid supplement in their drinking water as males. Posttreatment fecal samples for steatocrit were collected on day 64 and for coccidiosis on day 65. Birds were weighed and blood sampled again on day 66; they were released on day 68.

### 2.2 Acid steatocrit

**Figure 3:**
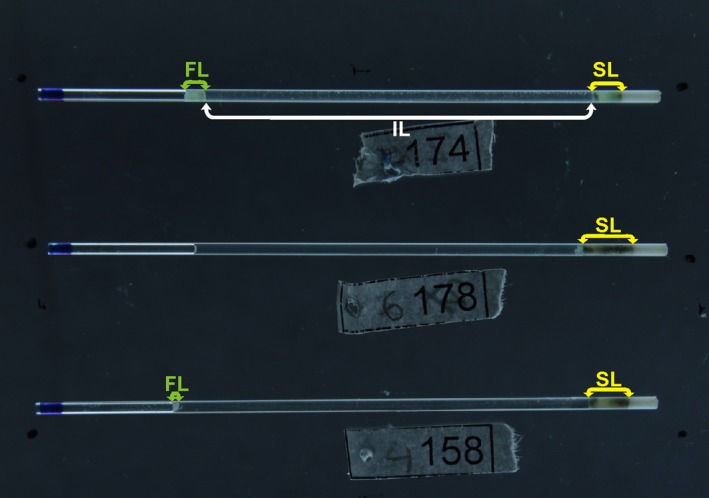
Nonfatty fecal solids layer (SL), liquid intermediate layer (IL), and fatty layer (FL). Upper hematocrit capillary tube shows a bird with severe steatorrhea; lower capillary tube indicates light steatorrhea. No fecal fat is visible in the middle capillary tube

Fecal fat content was estimated on the basis of the acid steatocrit method according to Tran et al. (1994). Bird droppings were diluted (1:3) with deionized water and homogenized. Five molar Perchloric acid was added in volume 1:5 to the homogenate which was subsequently vortexed for 10 s with a standard vortex mixer. The homogenate was collected into the hematocrit capillary tube which was then centrifuged at 13,000 rpm for 15 min. After centrifugation, the capillary tubes were photographed (Canon 1100D, 1s, f8, ISO100). The length in pixels was quantified for upper fat layer (FL) and solid bottom layer (SL) from the photographs (Figure 3) using ImageJ software. Steatocrit was expressed as a percentage of fat in the nonaqueous matter of the sample; that is, the length of a fat layer divided by the sum of the lengths of fat layer and solid layer. Spearman rank correlation coefficient between steatocrit values sampled in duplicate from the same fecal aliquot but processed separately was 0.71 (*p* = .0001, *n* = 24).

### 2.3 Statistics

Distribution of individual steatocrit values in males deviated from normality, also under various transformations (see Electronic Supplementary Material). We therefore applied nonparametric methods when analyzing steatocrit data. In order to test the effects of treatment on individual changes in steatocrit, Friedman ANOVA was applied separately for all treatment groups. Effects of treatments on body mass, plasma triglyceride content, and ln‐transformed coccidiosis intensity were analyzed in repeated measures ANOVA; residuals were normally distributed. Correlations between steatocrit and body mass, plasma triglyceride levels, and infection intensity were examined only in males because we had larger sample size and hence higher test power for males than females. Due to exploratory nature of the study, *p*‐values were not adjusted for multiple comparisons; alpha level of 0.05 was chosen as a criterion for significance. Sample sizes differ between analyses because one male bird died on day 5 (before the experimental treatments), one male bird from control group died on day 9, and one metronidazole‐treated male died on day 16. Blood samples for triglyceride measurement could not be obtained from all the birds.

## 3 Results

**Figure 4:**
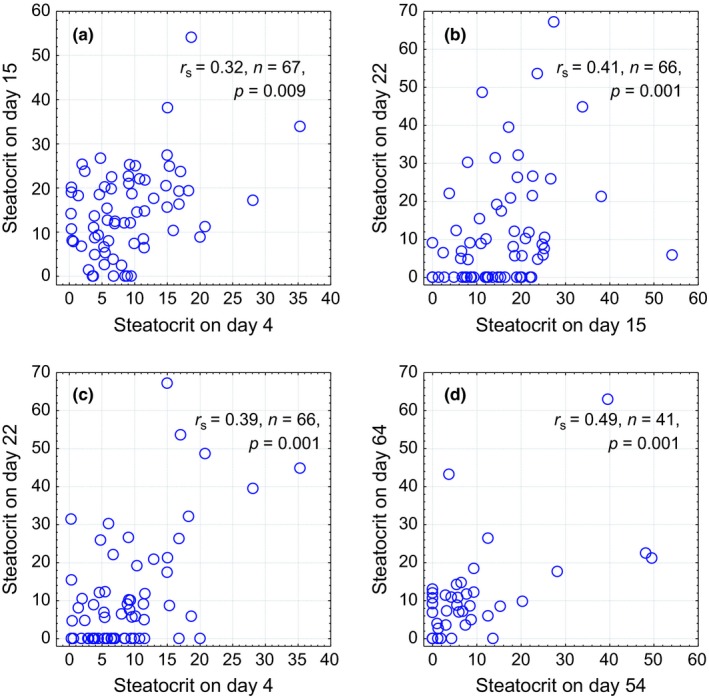
Spearman rank correlations and scatter plots between individual values of steatocrit, measured in different time points in male (a–c) and female (d) greenfinches

**Figure 5:**
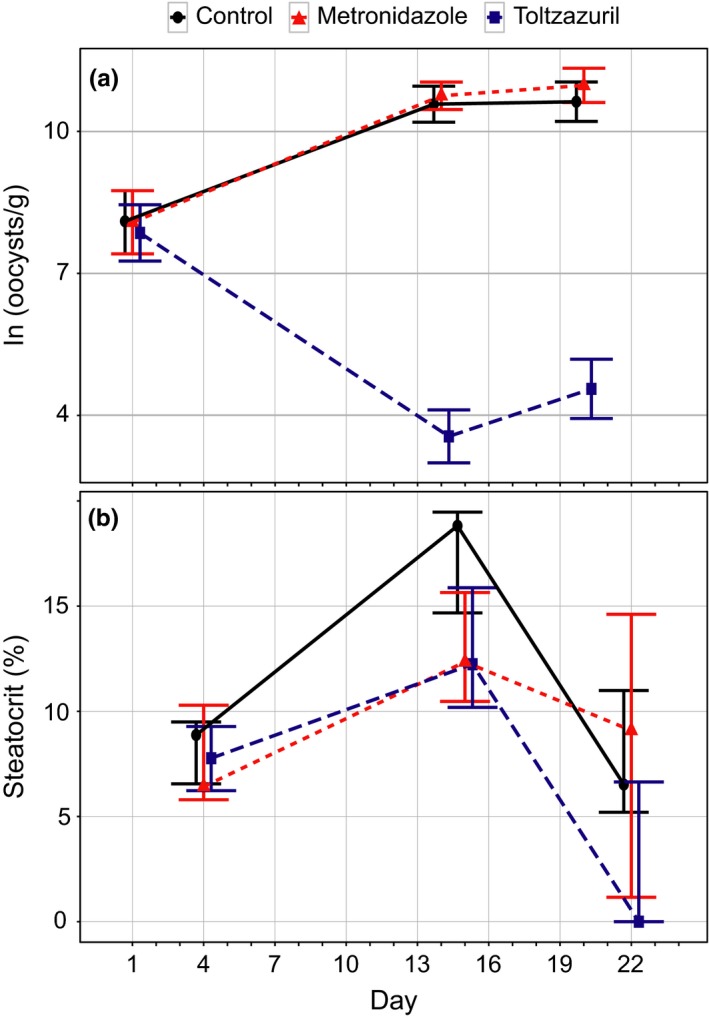
Effects of medication on intensity of coccidiosis (a) and steatocrits in male greenfinches. Means ± *SE* in (a), medians ± interquartile ranges in (b). Twenty‐two birds in control group and 23 birds in both medicated groups in the analysis of infection intensity. Twenty‐one birds in control and metronidazole group, 23 birds in toltrazuril group in the analysis of steatocrit. See Table 1 for *p*‐values for coccidiosis and text for steatocrit

Individual values of steatocrit showed significant individual consistency over 8, 12, and 20 days in males and over 11 days in females (Figure 4). In males, treatment with toltrazuril significantly reduced intensity of coccidian infection while metronidazole had no effect (Table 1, Figure 5a). None of the treatments affected changes in body mass (*F*_2,66_ = 0.1, *p* = .875) or plasma triglyceride content (*F*_2,47_ = 1.7, *p* = .191) on posttreatment blood sampling (*F* and *p*‐values for time × treatment interactions in repeated measures ANOVA). Metronidazole‐treated (χ^2^ = 2.3, *p* = .304) and control birds (χ^2^ = 4.9, *p* = .084) did not display significant variation in steatocrit over the three measurement occasions. Birds treated with toltrazuril showed reduced steatocrit values (χ^2^ = 10.1, *p* = .006) in the third measurement, that is, 8 days after the end of treatment (Figure 5b).

**Table 1:** Effects of treatments on intensity of coccidian infection on male and female greenfinches. See Figures 4 and 5 for the direction of the effects

Sex	Effect	*F*_*df*_	*p*
Male	Treatment	43.9_2.65_	<.00001
	Time	2.5_2.130_	.087
	Time×treatment	24.5_4.130_	<.00001
Female	Treatment	18.0_2.38_	<.00001
	Time	14.6_2.38_	<.00001
	Time×treatment	21.8_2.38_	<.00001

**Figure 6:**
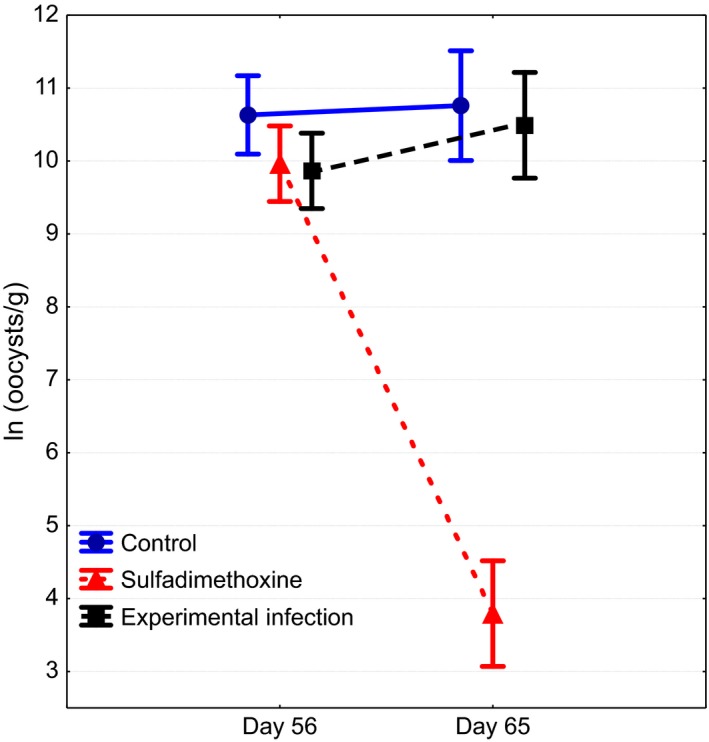
Effects of sulfadimethoxine treatment and experimental infection on intensity of coccidiosis in female greenfinches. See Table 1 for *p*‐values

**Figure 7:**
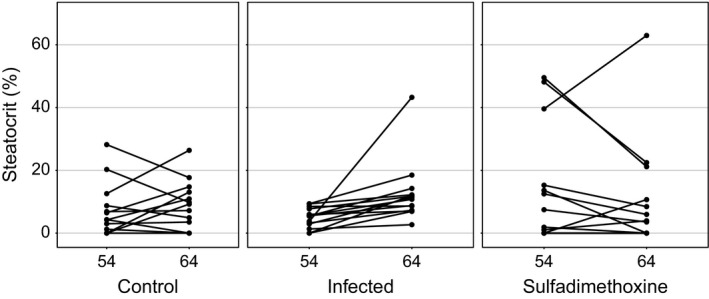
Individual changes in steatocrit values in female greenfinches in three treatment groups. Steatocrit increased in all experimentally infected birds (*z* = 3.3, *p* = .001, *n* = 14), no change in sulfadimethoxine‐treated (*z* = 1.2, *p* = .241, *n* = 14) and control birds (*z* = 0.6, *p* = .530, *n* = 13, Wilcoxon matched pair tests)

In females, treatment with sulfadimethoxine significantly reduced intensity of coccidiosis while no changes occurred in control and infected groups (Table 1, Figure 6). None of the treatments affected changes in body mass (*F*_2,38_ = 0.7, *p* = .569) or plasma triglyceride content (*F*_2,29_ = 1.1, *p* = .361) on posttreatment blood sampling (*F* and *p*‐values for time × treatment interactions in repeated measures ANOVA). Sulfadimethoxine‐treated (z = 1.2, *p* = .241, *n* = 14) and control birds (*z* = 0.6, *p* = .530, *n* = 13) did not display significant variation in steatocrit over the 11 days between the two measurement occasions (Wilcoxon matched pair tests). During the same period, by the 5th day after infecting, steatocrit increased in all experimentally infected birds (*z* = 3.3, *p* = .001, *n* = 14; Figure 7).

In males, intensity of coccidian infection on all three measurement occasions correlated positively with steatocrits measured on day 22, that is, 6 days after the treatments ceased (*r*_s_ = 0.24…0.31, *p* = .049… .009, *n* = 67). Steatocrit measured before the treatments, on day 4 predicted subsequent body mass loss between days 5 and 14 (*r*_s_ = −0.25, *p* = .043, *n* = 68). Birds whose plasma triglyceride levels declined between days 5 and 14 had increased steatocrits by days 15 (*r*_s_ = −0.33, *p* = .020, *n* = 49) and 22 (*r*_s_ = −0.29, *p* = .042, *n* = 48).

## 4 Discussion

Explaining why and how individuals differ in their performance is central to the evolutionary ecological research. This requires characterizing of individual phenotypic quality on the basis of biomarkers that are sensitive to experimental manipulations and individually consistent in time. This study found that digestive capacity assessed on the basis of steatocrit corresponds to such criteria. Values of steatocrit showed significant among‐individual variation but within‐individual consistency for at least 20 days, which means that some individuals were steadily better than others in digesting fat.

We tested the hypothesis that causes of individual fat absorption capacity were reliably captured by the steatocrit measure. Our experimental manipulations provided some insight about the causes of individual variation in fat absorption capacity. Male greenfinches that were administered toltrazuril against coccidiosis showed reduced values of steatocrit 7 days after the end of medication. Interestingly, there was no decline in steatocrit immediately after the end of medication period despite the significant reduction of oocyst shedding on day 15 (Figure 5). This suggests that repair of damage to intestinal surface, exerted by coccidians, takes at least a week in greenfinches. In 2‐week‐old chickens, the turnover time of epithelial cells is approximately 5 days (Fernando & Mccraw, 1973). It may thus appear that epithelial cell regeneration in adult passerines takes longer than in growing chickens. Such explanation would also clarify why we did not detect a decrease of steatocrit in sulfadimethoxine‐treated females that were sampled for steatocrit after 6 days of medication. It should be noted, however, that direct inhibition of fat absorption due to rupture of epithelial cells is just one possible pathway how coccidians can cause steatorrhea. Other possible mechanisms include impairment of intraluminal hydrolysis or solubilization of fat, decreased uptake of the products of fat hydrolysis by the epithelial cells, or abnormalities of mucosal cell transport (Sharma & Fernando, 1975). For instance, in turkeys coccidian infections that produce several weight depression and mortality can occur without much damage to intestinal mucosa (Madden & Ruff, 1979; Ruff, Augustine, & Madden, 1981).

Perhaps the most interesting finding of this study was detection of increase in steatocrit among the females infected with heterologous parasite strains. This treatment did not affect infection intensity, body mass, or circulating triglycerides, yet the effect on fat absorption was detected. This finding implies that steatocrit enables to identify much more subtle effects on digestion than measurement of oocyst shedding or circulating fats. It also confirms the previous finding in the same model system that new, heterologous parasite strains appear more virulent than familiar strains (Hõrak et al., 2006).

The finding that higher virulence (assessed on the basis of increased steatocrits) was obtained without eventual increase in infection intensities suggests that some parasite‐induced host response rather than the direct damage by parasites *per se* was responsible for the impaired fat absorption. Similarly, it has been observed in turkeys that decrease in absorption was not always related to the number of parasites in the cells or the extent of damage to mucosa (Ruff et al., 1981). One possible explanation for such patterns might be parasite‐induced reduction of host cell turnover time in the intestinal mucosa, so that the resultant shorter life of the epithelial cells might not permit normal differentiation into absorptive cells (Fernando & Mccraw, 1973). Experimental coccidian infection in chickens can also reduce the intestinal acidity and pancreatic production of digestive enzymes (Major & Ruff, 1978).

Differently from previous studies (Hõrak et al., 2004, 2006), we did not detect an effect of anticoccidian medication on plasma triglyceride levels or body mass. Notably, however, the steatocrit of males on the third measurement occasion correlated with infection intensity in all three measurements and changes in body mass and plasma triglycerides negatively correlated with changes in steatocrit. These correlations are consistent with a scenario that individuals who suffer more intense coccidian infection are inferior in digesting fat and that individual variation in fat absorption capacity eventually affects circulating triglyceride levels and body mass.

Our findings suggest that measuring steatocrit has an ample potential for application in ecological studies assessing effects of intestinal health on fitness‐related outcomes. For instance, intestinal and other infections often result in decline in body mass, but without measuring digestive capacity, it would be impossible to distinguish whether the effect of infection on body mass was caused by depletion of bodily reserves, reduced food intake, or malabsorption. Such studies would thus importantly contribute to understanding the mechanisms of how exactly parasites and pathogens impinge on fitness, a goal often not attained in immunoecological research (e.g., Schmid‐Hempel, 2003).

Although the utility of acid steatocrit has been successfully demonstrated in at least two species of mammals (see Introduction), we suggest that this method has particularly broad applicability in avian studies. For instance, unlike most of mammal species, birds possess tetrachromatic vision which enables extensive use of vivid integument coloration for signaling purposes (Hill & Mcgraw, 2006). Functions and mechanisms of such signals may thus largely depend on integrity of digestive machinery, which has so far been assessed only on the basis of distant endpoints such as circulating or deposited pigment levels. Similarly, it would be useful to test whether fecal fat content in free‐ranging birds, [in parallel with measurement of fecal parasite load and hormone metabolites (see e.g., Martínez‐Padilla, Redpath, Zeineddine, & Mougeot, 2013)] could be used for assessment of population health. Using steatocrit for quick diagnosis of fat malabsorption could be also of practical use for practitioners and veterinarians, including those working with endangered species in captivity.

To summarize, acid steatocrit is a simple, cheap, and noninvasive method for measuring of fecal fat content that can be used as a proxy for digestive efficiency in birds. In greenfinch coccidiosis, steatocrit appeared more sensitive to experimental manipulation of infection than plasma triglyceride levels, body mass, and in some occasions, infection intensity. This suggests that the method has wide applicability in avian ecophysiological research and calls for further testing of its use in different manipulations of intestinal health.
