# Supplementary figures and images for: Simple and noninvasive method for assessment of digestive efficiency: Validation of fecal steatocrit in greenfinch coccidiosis model
Source: Ecol Evol. 2016 Nov 17;6(24):8756–63. doi: 10.1002/ece3.2575 (PMC5192951; doi:10.1002/ece3.2575)

Electronic supplementary material ESM1. Distributions of individual steatocrit values.

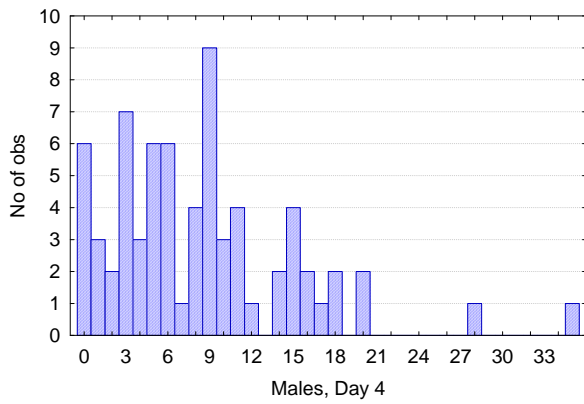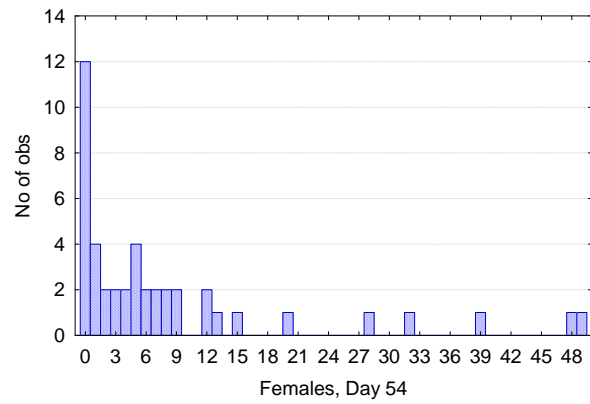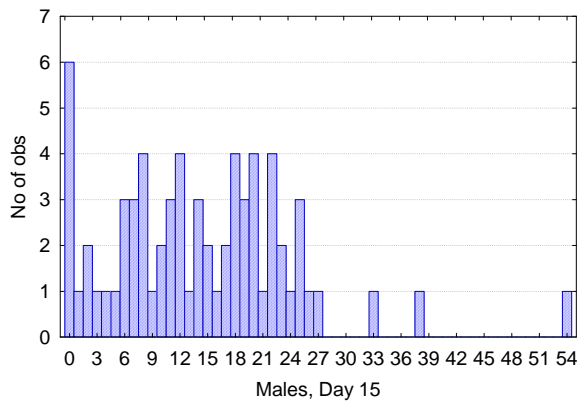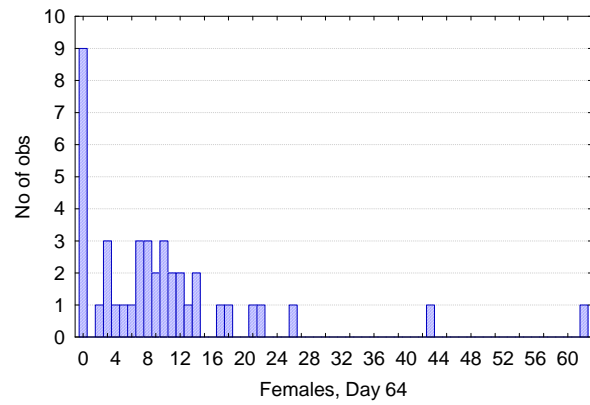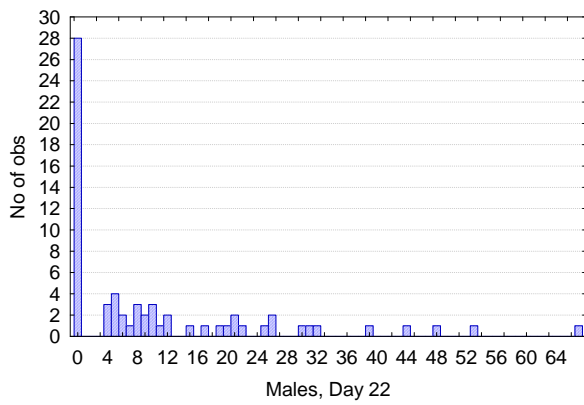

Supplement: Supplementary file 1 [file ECE3-6-8756-s001.pdf]
